# On the Determination of Mechanical Properties of Aqueous Microgels—Towards High-Throughput Characterization

**DOI:** 10.3390/gels7020064

**Published:** 2021-05-31

**Authors:** Ingrid Haga Oevreeide, Renata Szydlak, Marcin Luty, Husnain Ahmed, Victorien Prot, Bjørn Helge Skallerud, Joanna Zemła, Małgorzata Lekka, Bjørn Torger Stokke

**Affiliations:** 1 Biophysics and Medical Technology, Department of Physics, NTNU The Norwegian University of Science and Technology, NO-7491 Trondheim, Norway; ingrid.h.ovreeide@ntnu.no (I.H.O.); husnain.ahmed@ntnu.no (H.A.); 2 Institute of Nuclear Physics, Polish Academy of Sciences, PL-31342 Krakow, Poland; renata.szydlak@ifj.edu.pl (R.S.); marcin.luty@ifj.edu.pl (M.L.); joanna.zemla@ifj.edu.pl (J.Z.); 3 Biomechanics, Department of Structural Engineering, NTNU The Norwegian University of Science and Technology, NO-7491 Trondheim, Norway; victorien.prot@ntnu.no (V.P.); bjorn.skallerud@ntnu.no (B.H.S.)

**Keywords:** microgel, AFM, micropipette aspiration, high-throughput, mechanics

## Abstract

Aqueous microgels are distinct entities of soft matter with mechanical signatures that can be different from their macroscopic counterparts due to confinement effects in the preparation, inherently made to consist of more than one domain (Janus particles) or further processing by coating and change in the extent of crosslinking of the core. Motivated by the importance of the mechanical properties of such microgels from a fundamental point, but also related to numerous applications, we provide a perspective on the experimental strategies currently available and emerging tools being explored. Albeit all techniques in principle exploit enforcing stress and observing strain, the realization differs from directly, as, e.g., by atomic force microscope, to less evident in a fluid field combined with imaging by a high-speed camera in high-throughput strategies. Moreover, the accompanying analysis strategies also reflect such differences, and the level of detail that would be preferred for a comprehensive understanding of the microgel mechanical properties are not always implemented. Overall, the perspective is that current technologies have the capacity to provide detailed, nanoscopic mechanical characterization of microgels over an extended size range, to the high-throughput approaches providing distributions over the mechanical signatures, a feature not readily accessible by atomic force microscopy and micropipette aspiration.

## 1. Introduction

Mechanical properties of polyelectrolyte hydrogels are a fundamental characteristic that has been extensively addressed both experimentally and theoretically. Most of the approaches investigate bulk specimens of these soft materials. In the present perspective, we summarize efforts directed towards the determination of mechanical properties of small specimens in the form of microgels. The rationale for the focus on microgels is related to the large interest in exploiting polyelectrolyte hydrogel materials in the form of microgels both to address fundamental issues and in numerous applications. Furthermore, the microgel mechanical properties may differ from that of the bulk specimens of the same constituents either due to statistical variation of mechanical properties of microgels due to structural heterogeneity [1], combination of materials to yield multicomponent microgels different from that possible in bulk [2], coating of the microgels with the possible consequence of the mechanically stratified structure and immobilization of components in the microgels [3,4,5]. 

Microgels are already present in various domains serving as materials for developing lubricants [6], 3D bioprinting [7], 3D cell culture supports [8], sensors [9] and plugging of porous media [10]. Microgel properties such as size and size distribution, colloidal stability, swelling, environmental responsivity, and mechanical properties are important for such applications. These properties can be designed and tailored by their synthesis conditions, including choice and concentration of monomer, co-monomer(s) and crosslinking agents. The various concentrations of the components and the distribution of crosslinking sites are the most important parameters influencing the mechanical properties of the microgels. Thus, the understanding of how mechanical properties vary in response to the type of the solvent, its composition, the presence of divalent ions and the concentration of crosslinking agents is of great importance. 

**Figure 1:**
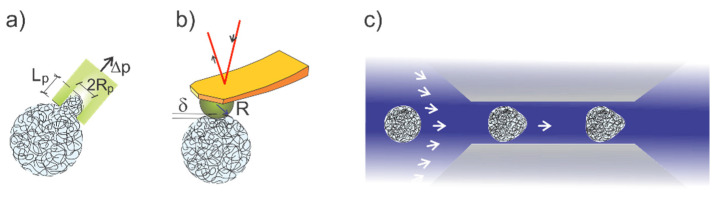
Schematic illustrations of micropipette aspiration (**a**), atomic force microscopy-based nanoindentation (**b**) and squeezing in microchannel confinement (**c**) for characterization of mechanical properties of microgels. In micropipette aspiration (**a**), a suction pressure (Δp) applied by a micropipette with inner radius R_p_, in contact with the microgel leads to suction with length L_p_ of part of the microgel in the pipette. Analysis of experimentally determined L_p_ as a function of Δp combined with appropriate material model yields an estimate of mechanical properties; (**b**) Schematic illustration of colloidal bead (radius R) tip geometry on an AFM cantilever indenting a microgel with total indentation δ; (**c**) Illustration of microgels in a fluid stream in a constriction channel with cross-sectional dimensions larger than the microgel being deformed due to the distribution of stress imposed on the microgels by the flow field.

In the following, we summarize conventional strategies for determining the mechanical properties of aqueous microgels. This includes techniques such as micropipette aspiration [11,12], optical tweezer and stretcher [13], atomic force microscopy [14,15,16], and, more recently, microfluidic-based approaches to provide distribution of the mechanical properties [17,18,19], and also Brillouin scattering based imaging [20]. Schematic presentations of some of these strategies are shown in Figure 1. Microgel mechanical properties can also be deduced from methods such as inferred from calorimetry [21], magnetic microdisc rheometer [22] or bulk rheological measurement of close-packed microgel dispersions [23]. Most of the approaches share the principle of analyzing deformation—stress relations for the actual physical realization of the experiments, which may also include explicit material models and distribution of the mechanical properties. We thus summarize relevant aspects as underpinning the various techniques, targeting both a basis for the analysis as well as a comparison between the different experimental approaches. An important part of this is possible differences in the rate of deformation/stress, or frequency for the corresponding cyclic experiment for the obtained mechanical properties. Thus, awareness of the dynamics of the obtained mechanical properties is an essential facet in the understanding of the aqueous, including polyelectrolyte microgels as well as in the comparison of the experimental approaches.

The paper is organized in the following by first giving examples of relevant experimental preparation approaches exploited for the fabrication of microgels; then methods not supporting high-throughput characterization such as atomic force microscopy and micropipette aspiration are presented. This is followed by summarizing high-throughput strategies as, e.g., supported by flow techniques in microfluidic channels before also alluding to some emerging approaches for the field. Relevant examples are included alongside the discussions of the individual techniques, and the perspective is concluded by comparing the various approaches. 

## 2. Fabrication of Microgels

A brief account of strategies employed for the preparation of microgels is included since this is required for any subsequent mechanical characterization unless the sample occurs naturally. Generally, the preparation of micro- and nanogels is realized using a range of materials and strategies as recently reviewed [24,25]. Microgel synthesis can be stated to require a monomer, crosslinker and initiator, for either of the two main preparation routes being based on emulsions in bulk or as supported by microfluidic approaches. Several different techniques can be employed for the formation of such microgel spheres. These microgels are typically spherical and with a diameter scaling from 100 nm to several hundred micrometers. 

There are several different strategies that can be employed for bulk preparation of microgels, such as precipitation polymerization, spray drying, membrane emulsification and using template molds (Table 1). The chosen strategy will depend on the microgel properties and applications, as these will determine the appropriate use of solvents, UV-light, temperature and pH-initiated polymerization processes. Controlling the ratios of the components as well as temperature and pH may offer strategies to affect the size, shape, polydispersity, elasticity and overall microgel yield [26]. 

Microgel elasticity is tuneable through the selection of composition, and crosslinker concentration, and can also be affected by temperature and hydration levels. The addition of low concentrations of crosslinker resulted in the formation of ultralow crosslinked microgels, which can be further used as biomimetic particles. Muller et al. utilized ultrasound to enhance microgel deformation, as controlled deformation can be desired in several biomedical applications, such as drug delivery. The deformation of such microgels has also been observed to influence their biocompatibility [27]. Increasing the crosslinker (PEGDMA) concentration from 1% to 11% resulted in a change in shear modulus (G’) of the microgel from 1.1 ± 0.1 kPa to 17 ± 1 kPa [28]. A similar effect was also observed when crosslinker PEGDA was added at 1 and 10%, resulting in the modulus increasing from 7.8 ± 1 kPa to 63.9 ± 15.7 kPa [29]. Thus, the change in mechanical properties of these microgels also depends on the type of crosslinker implemented.

The elasticity of single microgel particles was determined when the particle underwent a deswelling process due to temperature changes [30]. In this instance, an increase in temperature from 300 K to 313 K resulted in both a decrease in the size and a corresponding increase in Young’s modulus from 8 ± 1.4 kPa to 86 ± 22 kPa. Such microgels have also been used in regenerative medicine. In one instance, a doubly crosslinked microgel was injected into degenerated intervertebral discs to improve the mechanical properties. Subsequent cell mobility studies in matrices prepared by embedding the microgels in a fibril polymer network were shown to vary the cell migration rates depending on the elasticity of the microgels [31].

As illustrated by the examples above, the mechanical properties of microgels are essential in biological applications, from the proliferation and viability of cells to microgels’ migration in physiologically relevant environments. A key facet here is the cells’ ability to sense the elastic/mechanical properties of their environment and they can respond to this in a chemical manner which can then change the cell properties and thus also their behavior [32]. Therefore, it is important to measure and know the mechanical properties of the different microgels that can be produced, especially within this field.

Microfluidic-assisted hydrogel beads’ fabrication is another synthesis route, possibly offering more explicit control of the resulting size of near monodisperse samples than the above summarized methods. The microfluidic approach is widely exploited due to its ability to provide three-dimensional support to biological entities since it also allows their inclusion in the process [42,43]. Fabrication of microgels using this route hasbeen reported for various polymers using either physical or chemical gelation strategies (Table 2 and [44,45]). The chemical gelation methods mostly induce the gelation of synthetic polymers such as PEG and acrylamides by photoinitiated or redox-initiated polymerization, which are synthesis strategies also employed in bulk approaches. When selecting polymers and crosslinking method for the microfluidic-assisted fabrication of microgels, consideration of facets such as, e.g., crosslinking method, compatibility of the polymer with end-use of the microgels, ease of emulsification and compatibility with encapsulation capacities, represent a basis for the actual selection. With respect to crosslinking, the example of the production of Janus microgels by applying UV radiation on an aqueous emulsion of monomers to initiate the chemical reaction [18,46,47] illustrate the need to consider adverse effects of the crosslinking process. This method produces mechanically strong microgels, but UV radiation could be harmful to biological molecules. 

Ionic gelation can generate microgel beads by on-chip emulsification at the pregel state followed by coalescence of aqueous droplets including the crosslinker, e.g., aqueous sodium alginate and crosslinking agent (CaCl_2_). Diffusion of a crosslinking agent such as Ca^2+^ or Fe^3+^ ions from continuous phase to emulsified precursor has also been suggested [48]. Among the various approaches reported (Table 2), some of the them require a change of pH in the post-emulsification region, whereas others proceed at constant pH. The challenge associated with the reduced pH in the examples with alginate microgels has been addressed in the method of competitive ligand exchange reaction [49]. This approach exploits a gelling ion-chelator and exchange ion-chelator combination that satisfy the order of association constants for a cascading exchange when the two streams meet just before the emulsification in a flow-focusing device. While stable and non-reactive before being blended, the merger of the two aqueous streams induces the release of the gel-inducing ions by a displacement from the exchange ion. This cascading process proceeds at constant pH compatible with living cell requirements.

While the microfluidic-assisted approaches provide excellent control of mean population size and mono-dispersity of synthesized microgel samples, e.g., as illustrated by the polymer particle size distributions reported for microfluidics route as compared to the bulk emulsification [55], there are current limitations in throughput [4] that should be relaxed to provide a larger amount of samples. Thus, devices supporting massive multiplexing for droplet generation demonstrated for aqueous phase with viscosity up to 155 mPas represent an interesting innovation that could pave the way for enhanced hydrogel bead fabrication rates [56]. Another approach is the in-air-microfluidic approach demonstrating a 10–100 fold improved fabrication rate of microgel-like particles compared to the microfluidic device-based microgel synthesis and also maintaining capabilities such as fabrication of Janus microgel particles [57,58]. Overall, there is a wide range of described methods that can be exploited to synthesize a particular microgel, offering versatility in polymer compositions and their distributions, that also allow for immobilization of living organisms [59]. This is an important toolbox underpinning sample design and realization of polyelectrolyte microgel samples for mechanical characterization.

For the majority of bulk fabrication processes, there are extensive purification and washing steps involved, as there are often uncreated reagents present in the bulk emulsion after reaction completion; thus, increasing the time and complexity of the overall microgel process compared to microfluidic processes. This is exemplified by the need for micro sieves with mesh sizes ranging from 40–630 µm showing overall low control of particle dispersity [28], or the need for removing unreacted reagents over a 5-day period [36]. Thus, biocompatibility, degradability and stability to external variations are also factors that must be considered when selecting an appropriate fabrication procedure. 

## 3. Application of AFM to Determine Mechanical Properties of Microgels

Atomic force microscopy (AFM) is a widely used technique employed to quantify the mechanical properties of various soft materials, including hydrogels [60], microgels [61] and living cells [62]. In the AFM, the sample is attached to a support placed, typically, on a piezoelectric *XY* scanner. A cantilever with a probing tip mounted at the free end of the cantilever deflects due to forces acting between the tip and the sample surface. To detect the deflection, typically, an optical system is used, in which a laser beam is focused on the cantilever free end, just above the probing tip. The reflected laser beam is registered by a position-sensitive photodetector (a photodiode). The active area of the detector is, typically, divided into four quadrants, two top and two bottom ones. The forces acting perpendicularly to the investigated surface move the cantilever up and down what translates to the difference between top and bottom quadrants, expressed in volts. The difference is converted into force by multiplying it by the cantilever spring constant (N/m) and photodetector sensitivity relating volts with nanometers. The advantage of the AFM is the capability to carry out the measurements in liquid conditions making this technique particularly suitable for measuring microgels in the actual solvent conditions. As required by the AFM method, the microgels had to be first adsorbed/adhered at a solid surface to render mechanical testing reliable [14]. AFM working in force spectroscopy mode delivers the force curves (relations between the force and the relative scanner/sample position) measured on the stiff, non-deformable surface and soft material. By subtracting these curves from each other, a force versus indentation depth curve is obtained. Such a curve is a basis for theoretical models describing the deformation of elastic, isotropic material by a rigid, axisymmetric indenter such as the widely used Hertz-Sneddon contact mechanics. The Hertzian contact mechanics yields the following relation for the force, *F*, versus indentation depth, δ, for a spherical indentation geometry (radius *R*) probing a homogeneous elastic medium:

(1) $$F=\frac{4}{3}\frac{E\sqrt{R}\;}{1-\nu^{2}}\delta^{3/2}$$

where *E* is the material’s Young’s modulus and ν is the Poisson ratio. Here, it is assumed that the Young’s modulus of the indenter is much larger than the material probed. Overview of the impact of indentation geometries and elaborations of the theoretical basis for analysis of nanoscale mechanics has been provided [63], and also, more recently, summarizing, e.g., operation in dynamic modes [64]. Here we have chosen to focus on applying AFM to characterize aqueous microgels, including also samples made of polyelectrolytes [65,66,67,68,69,70]. AFM stands apart from most methods for determining the mechanical properties of microgels since it offers information at the nanoscale. Several studies have used AFM to directly measure the properties of individual microgel particles, focusing on the impact of their internal structure [61,71,72,73]. Mechanical properties of microgels depend on many parameters, which generally can be grouped into external factors and internal parameters associated with synthesis conditions. Temperature, pH and solvent composition have been identified as important external factors affecting the mechanical properties of microgels [74,75]. In Table 3, selected examples of the application of AFM for the determination of mechanical properties of microgels are summarized. 

Results reported on the widely studied microgels made of poly (N-isopropylacrylamide) (PNIPAM) are included as an example of a material displaying inherent responsive character to various external stimuli [26]. AFM studies demonstrated that the crosslinker affects the swelling behavior and elastic properties of PNIPAM microgels [74]. With the increasing amount of crosslinker, the microgel ability to shrink decreases during the heating and cooling processes. The height/width ratio of individual microgel particles was preserved for high crosslinker concentrations, while at the low concentrations, the shrinking and swelling was accompanied by changes in microgel height. Moreover, the center of the microgel became more rigid with increasing crosslinker concentration and temperature. In another report, the mechanical properties of PNIPAM microgels were determined as a function of the crosslink-monomer ratio [61]. Alongside the observed increased rigidity of the microgels as the ratio became higher, it was possible to obtain a radial distribution of mechanical properties. The microgels’ core was stiffer (17 to 50 kPa) than its peripheral (3 to 40 kPa) parts, which was a difference elucidated for regions of the microgels in order of tens of nm apart.

Moreover, highly crosslinked microgels seem to be more homogenous in terms of mechanical properties. The microgels are sensitive to temperature. The AFM-based elasticity measurements, conducted on P(NIPAM-co-AAc) microgels, show temperature-dependent microgels’ response (their shrinking) in an aqueous solution above their volume phase transition temperature (VPTT). Using various mixtures of two good PNIPAM solvents, i.e., water and ethanol, demonstrated that the interaction between polymer and solvent affected the rheological properties of microgels [72]. An interesting example of the AFM measurements are microgels whose swelling is governed by electrostatic interactions. The peak-force quantitative nano-mechanics (PF-QNM) measurement mode applied to poly(ethyl acrylate-co-methacrylic acid) microgels revealed that the mechanical properties depend on the available fraction of charged groups present within the microgels, related to charge density. Interestingly, the microgels of intermediate charge densities were more deformable [75]. In one of the early studies, the mechanical properties of hydrogels’ microspheres were measured to estimate the resistance to compression affected by chemical composition [65]. The results show that all alginate-based hydrogels exhibited pure elastic behavior with elastic (Young’s) modulus ranging from 0.4 to 440 kPa. Microspheres containing BaCl_2_ appeared to be softer than those containing CaCl_2_. In more recent studies, the coating of alginate microspheres with genipin-crosslinked double poly-L-lysine (PLL) results in a tighter organization of poly-L-lysine crosslinked by genipin [76]. More advanced AFM working modes such as the high-speed AFM (HS-AFM) can also be involved in the characterization of microgels. In one example [80], HS-AFM was applied to study the real-time adsorption of various types of microgels to verify the hypothesis that the microgels’ deformability affects the adsorption’s kinetics to a solid surface in aqueous conditions. Results show that more deformable microspheres adsorb faster than more rigid ones, thus, showing the possibility to design microgels characterized by the specific adsorption properties.

Overall, these examples from the application of AFM for the determination of mechanical properties of microgels highlight that rich, microgel-specific signatures can be obtained. Furthermore, related to a more practical side in performing such experiments is the variety of immobilization strategies employed, which can serve as a source of inspiration when embarking on new samples.

## 4. Micropipette Aspiration for Microgel Mechanical Characterization 

In the micropipette aspiration technique, a micropipette is brought in contact with the surface of the deformable object, and a small suction pressure applied through the micropipette is generating the stress field leading to the suction of the soft material into the pipette (Figure 1a), which is typically determined using conventional optical microscopy. Combining the experimental data of applied pressure difference and suction length with the appropriate material model yields an estimate of the mechanical properties. Thus, the suction of a soft material modeled as homogeneous in half-space yields the following relation between the pressure difference Δ*p* and aspiration length at the central micropipette symmetry axis, *L*_p_ [81]:

(2) $$\Delta p=\frac{2\pi EL_{p}}{3R_{p}}\Phi$$

where *E* is Young’s modulus, *R*_p_ is the micropipette radius and Φ is the wall function determined by the geometry of the pipette, and a value of Φ = 2.0–2.1 [82] is commonly used. Zhou and coworkers extended the analysis of the mechanically homogeneous case to micropipette suction of spherical reference state using a finite element approach [83]. Within a range of pipette radii relative to the radius of the spherical reference state, *R*_c_, they provided the based on non-linear regression to the FEM results: following empirical relation

(3) $$\Delta p=\frac{E}{3}\frac{L}{R_{p}}\left[\beta_{1}+\beta_{2}\frac{L}{R_{p}}\right]\left[1-{\left(\frac{R_{p}}{R_{c}}\right)}^{\beta_{3}+\beta_{4}\frac{L}{R_{p}}+\beta_{5}{\left(\frac{L}{R_{p}}\right)}^{2}}\right]$$

where the coefficients were β_1_ = 2.0142, β_2_ = 2.1186, β_3_ = 2.1187, β_4_ = −1.4409 and β_5_ = 0.3154. Thus, the basis for the analysis of the pressure-aspiration length profile will yield an estimate of Young’s modulus that is less for the half-plane theoretical expression than that based on the suction of the spherical bead, and the deviation becomes larger, the smaller the *R*_c_/*R*_p_. 

The micropipette aspiration technique has also been extended to map dynamic properties applying a cyclic pressure gradient [12]:

(4) $$p\left(t\right)=p_{0}\;sin\left(\omega t+\theta_{P}\right)+const$$

and determining the resulting aspired length change:

(5) $$L\left(t\right)=L_{0}\;sin\left(\omega t+\theta_{L}\right)+const$$

Following standard approaches in periodic loading experiments, the dynamic storage, *E*’, and loss, *E*’’, moduli were determined from the amplitudes and phases:

(6) $$E^{\prime }=\frac{3R_{p}\Phi \;p_{0}}{2\pi L_{0}}cos\left(\Delta \theta \right)$$

(7) $$E^{\prime \prime }=\frac{3R_{p}\Phi \;p}{2\pi L_{0}}sin\left(\Delta \theta \right)$$

where Δ*θ* is the phase difference Δ*θ* = *θ*_P_ − *θ*_L_ between the phases of the applied pressure and resulting aspired length, *p*_0_ and *L*_0_ are the amplitude of the cyclic aspiration pressure and length, and ω is the frequency.

Examples of mechanical data reported for microgels employing micropipette aspiration strategies include determining Young’s moduli of gelatin microgels of various sizes as gelled in a confined space made of liposomes [84]. They reported an increase in *E* when the radius of the microgels was less than ~40–50 µm, and combined with other characterization tools interpreted the increased elasticity in view of differences in the extent of higher-order structures existing in the droplet approach as compared to the bulk state. Thus, the increased amount of β-sheet in the microgels, suggested to be due to a confinement effect, was suggested to be the structural basis for the increased elastic modulus. The extension to determine dynamic elastic properties of microgels, also in the case of gelatin, resulted in the identification of both an increased storage modulus and decreased loss modulus of the material in the microgel form as compared to the bulk state. Stöver et al. [85] employed micropipette aspiration to determine mechanical properties of Ca-alginate hydrogel beads, their subsequent coating with polycation layer and treatment of resulting structures with citrate to reduce the extent of Ca^2+^ mediated crosslinking of alginate in the core. Examples of results obtained include reduced *E* as a function of extended storage of the alginate microgels, and changes in apparent Young’s modulus associated with the transformation of initial alginate microgel bead to a capsule, with a more viscous core. In this approach, the theoretical assumption of a homogeneous elastic body being interrogated by the micropipette aspiration strategy underpins the reported data. 

While examples of micropipette aspiration to determine mechanical properties of microgels highlight the possibility to extract information identifying specific features related to microgels, such as confinement and impact of transformation from a microgel bead to a capsule, there appears to be potential for improvements in the approaches. This includes the application of results such as explicit consideration of the deformed object being spherical in its reference state and more realistic distributions of mechanical properties.

## 5. High-Throughput Microgel Mechanics’ Determination in Microchannels

While the methods presented above currently represent core tools in assessing mechanical properties of microgels, scanning probe and micropipette aspiration are generally considered as not being ideal for rapid characterization of a large number of microgels at the individual object level. Thus, techniques based on feeding microgel objects in devices, subjecting them to mechanical characterization mimicking strategies in flow cytometers, have been developed. Techniques exploiting squeezing of microgels in tapered microchannels, forcing them through constrictions or exposing the soft entities to fluid shear stress or elongational stress, one at a time, have been reported. In the case of microgels passing through constrictive microfluidic channels, several analytical mechanical models have been proposed [86,87,88] to define relations between the measured pressure, the radius ratio of the elastic sphere to the channel and the global elastic property of the microgels, thus underpinning the analysis of experimental observations. These models are limited to microfluidic channels with a diameter smaller than an unperturbed microgel size. In one example, the deformable microgels are fed into a tapered microchannel. When transported into increasingly narrower parts by a liquid flow, the microgels will eventually block the channel for the liquid to pass, resulting in a situation where the elastic deformation of the microgel will balance the pressure difference. However, the modes proposed by [86,87] cannot be applied to high- throughput techniques.

Forcing soft entities through even microchannels with cross-section less than the cross-sectional area was introduced to determine mechanical features of cancer cells (constriction-based deformability cytometry, cDC) [89], and can also be applied for the determination of microgels. In this approach, the passage time through a given length of the constriction is used as an indicator of mechanical properties [90,91]. Other approaches include deformation induced in the stagnation point in opposing channels in devices supporting elongational flow (extensional deformation cytometry, xDC) [92]. In this case, the mechanical signature is estimated as the ratio of the long and short axis of the deformed object as determined by optical imaging. Shear stress induced in a constricted channel, but with a cross-section larger than the object to be deformed (shear deformation cytometry, sDC) is a third approach to high-throughput characterization applicable for microgels. In their report, Mietke and coworkers used a deformability parameter extracted from images captured by a high-speed camera and combined this with analysis of the deformation of mechanically homogeneous objects as well as structures with a shell [93]. 

**Figure 2:**
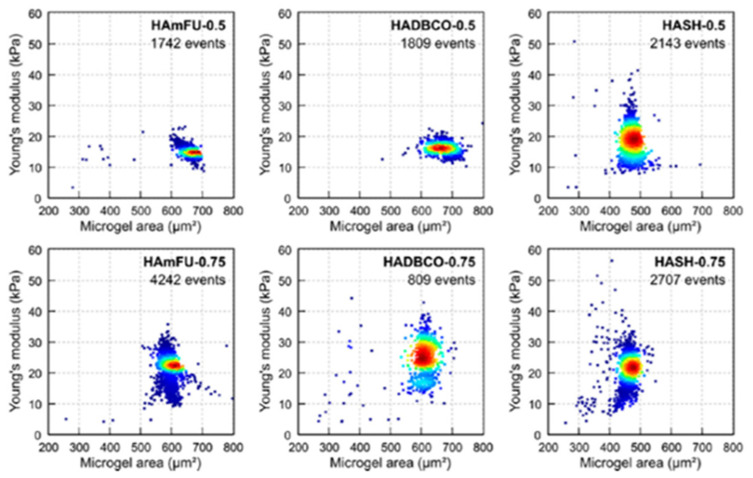
Scatter plots of mechanical properties of various hyaluronic acid derivative microgels determined by RT-DC. The intensity maps of Young’s modulus versus cross-sectional area of the microgels are shown for three different types of HA derivative microgels, each at two concentrations of PEG-based crosslinker. The RT-DC characterization was reported for characterization of in a microfluidic channel with 40 × 40 µm^2^ cross-section and 300 µm long using a flow rate of 0.60 µL s^−1^ [18].

These toolboxes underpinning real-time measurements of microgel deformation provide high-throughput at different rates, and impose stress at different rates [94]. The awareness of the latter differences is important when comparing different tools to determine the moduli since the materials may have intrinsic frequency dependent properties. The development of these deformability cytometry (DC) tools, originally intended for the characterization of cells, has also migrated to the measurements of microgels. More recent advancement of the technique includes modes such as real-time-DC (RT-DC), quantitative-DC, or dynamic RT-DC (dRT-DC) [17,95]. Spherical PNIPAM microgels with two different crosslinking densities characterized by such DC techniques were found to produce egg-like to triangular-shaped deformed states at a shear rate of 163 s^−1^, which was deformed further to parachute-like structures at 326 s^−1^ for the specimen with 0.15 wt% crosslinker. The focus was on the changing capillary number on the deformation behavior of microgels at two different crosslinker concentrations. The specimens in this study were generated using a flow-focusing strategy realized by a microfluidic device, ensuring a relative standard deviation of 7.7% of the 249 µm diameter microgels. While the highly desirable, nearly monodisperse character of microgels subjected to DC and RT-DC characterization was realized in this study, the actual microgel sizes were larger than that of cellular structures. Although not being a polyelectrolyte hydrogel, the previous example, and the reported RT-DC determination of polyacrylamide hydrogels with a diameter less than 20 µm [17] illustrate features of this technique. In their report, Guck and coworkers [17] prepared polyacrylamide (PAA) microgels being close to 12 µm diameter and nearly independent of monomer concentration in oil (all with relative standard deviation in the interval 5.6 to 8.3%) before being transferred and equilibrated in aqueous solution. The PAA microgels increased in size, dependent on the composition following equilibration in an aqueous solution. The RT-DC measurement yielded Young’s moduli for the microgels of mean value in the range 1.2 kPa to 1.6 kPa for different batches of the sample with 7.9% polymer concentration, and a relative standard deviation of Young’s modulus between 11 and 21%. While the authors reported overlaps in the range of *E* determined with RT-DC and AFM, it is interesting to note that the relative standard deviation of the microgels determined by AFM in the range 23–51% is larger than observed when using RT-DC. Another noteworthy feature reported in this study is the relaxation time of 0.12 ± 0.02 ms for the deformation of the microgels, a parameter that was deduced from the analysis of images of deformed states along the channel. A further example of RT-DC applied to microgels is the analysis of covalent crosslinked hyaluronic acid (HA) microgels aided by flow focusing in obtaining nearly monodisperse microgel samples, with a mean diameter in the range 24 to 30 µm, depending on actual HA modification and crosslinking chemistry, as equilibrated in phosphate-buffered saline [18]. The RT-DC provide distributions of the mechanical properties (Figure 2) that show a mean value of Young’s modulus that increases with increasing crosslinking density and depends on the derivatization strategy. Moreover, the heat-plots also indicate that the smaller fractions of the hydrogel samples have a significantly larger Young’s modulus than the main trend within the sample. This indicated that the technique might serve as a viable strategy to determine subfractions with altered mechanical properties in a label-free strategy.

In many of the approaches overviewed above, analysis strategies routed in analytical models are used as a starting point for, e.g., the real-time classifications of soft objects. These analytical models neglect the interaction between the deformed body shapes and changes in the surrounding hydrodynamic flow profiles. Various research has demonstrated that analysis of mechanical properties of cells results in large variability of two main origins, namely, biological variability and technical inaccuracy. The use of microgels as the mechanical and geometric model of the cells allows for the development of protocols related to measurements, data analysis and interpretation to be applied to cells. Recently, numerical models that can account for the interaction between the fluid and deformable cells in microfluidic devices have been developed to interpret the results of high-throughput experiments. These numerical models show good potential for cell stiffness assessment from high-throughput microfluidic devices as they can account for various channel geometries and flow conditions. Belotti et al. [96] presented a fully-coupled time- dependent fluid-structure interaction (FSI) finite element model of a hydrodynamic stretcher using COMSOL Multiphysics. Saadat et al. [97] reported an immersed boundary (IB) method to model red blood cells moving in microfluidic constriction larger than the cells. Mokbel et al. [98] reported a grid-based FSI model implemented in ADMis [99] applied to eukaryotic cells in microfluidic constriction larger than the cell.

While the examples summarize both the microgel and cellular specimens, the high- throughput strategies often employ the characterization of microgel beads for calibration that enables the classification of the measured cells. The simultaneous use of many channels to analyze deformations of breast and pancreatic cancer cells of different metastatic potential revealed that the higher metastatic potential of breast cancer cells correlates to higher deformations. In contrast, pancreatic cells revealed the opposite behavior [100]. Recent studies have shown that Fourier analysis of cellular-shape modes allows disentangling the cell response to time-dependent and time-independent hydrodynamic stress distributions [95]. These examples underline the usefulness and applicability of microgels in developing biorheological/biomechanical-based methods to analyze the properties of pathologically or functionally altered cells. 

## 6. Local Microgel Deformation by Optical Techniques

Various optical strategies beyond those integrated, e.g., for image acquisition, have also been discussed for application to determine mechanical properties of microgels. Application of molecular rulers functionalized with donor-acceptor fluorophore pairs supporting Förster resonance energy transfer (FRET) is an emerging strategy that has been applied for reporting changes in proximity between various entities, in particular, associated with cell adhesion [101], but also included as molecular reporters of changes in local deformation in microgels [102]. The fact that FRET provides information on the proximity within the donor-acceptor pair calls for calibration to transform the signal to mechanical stress. In this perspective, Thiele and coworkers combined AFM with confocal microscopy to apply a local, controlled force and observe the resulting change in the FRET signatures [102]. Thus, the local acceptor/donor intensity ratio was found to increase in the region adjacent to the contact area of the AFM-indented region, thus indicating that also local features (limited by the resolution in the employed microscope) can be determined. 

Thiele and coworkers [20] reported Brillouin shifts of hyaluronan derivatives’ microgels and found that the crosslinked microgel using a longer chain length of the bifunctional crosslinker yielded a larger Brillouin shift. This difference was stated to be parallel to trends in Young’s modulus observed by indentation of the same specimens. The Brillouin light scattering is a noncontact imaging technique that does not require contrast agents or labeling [103]. The probing mechanism involves the coupling of photons to longitudinal phonons and the obtained scattering spectra can be interpreted as the response of the sample to an infinitesimal uniaxial compression, and formally described by the longitudinal modulus (i.e., the compressional stiffness). The reported data by Thiele and coworkers on the Brillouin shifts [20] that can be translated to longitudinal moduli are considered interesting to gain more experience on the applicability of this tool to microgels, e.g., soft materials with a dominating fraction of water, for which the particular signal has been discussed [104,105]. 

## 7. Conclusions

A range of various approaches has been applied to determine the mechanical properties of microgels, including also those synthesized using polyelectrolytes. Although numerous interesting facets have been reported, the three main issues of awareness for work in this field appear evident. The first is related to the fact that differences in the rate of deformation/stress exposure which are not always explicit in the literature, may lead to difficulties in comparing results obtained using different strategies. Using standard theory for the frequency dependence of mechanical properties of polymers also in the crosslinked, stated as in microgels (e.g., [106]) as inspiration, differences in moduli at different frequencies are expected. Secondly, the impression that different levels of refinement of theoretical description are used to extract material parameters from the imposed and observable parameters is a further complication when comparing different approaches. A third facet to be aware of is the possible difference in mechanical properties of the microgels as compared to the bulk state of the same constituents. 

The mechanical properties in the macroscale rarely overlap with those measured on the nanoscale. This highlights the need to find and unravel the relation between microgels’ formation and/or surface functionalization with the structure, shape and mechanical properties. Understanding how mechanical properties of microgels vary at different length scales, and in response to the type of the solvent, composition and the presence of divalent ions, is of great importance in some mechanical properties of cells. Detailed characterization of microgels prepared with controlled constituents may therefore also serve as a route to understand changes in mechanical properties of cells. Microgels can model cell shape and mechanical properties. Understanding how these properties change is particularly significant in controlling, regulating and functioning of the cells. Biomechanical properties change during cancer progression. Cells became more deformable, as has been shown by AFM measurements conducted for various cancers, including bladder [107], lung, cervical and breast cancers [62]. 

Alterations of mechanical properties are not exclusively observed for cancer cells. Moreover, normal cells change their mechanics in response to altered conditions of the surrounding microenvironment. For example, endothelial cells exposed to chronic hyperglycemia [108] adapts their mechanical properties leading to dysfunction of the endothelium [109]. In simpler cells such as bacteria, the wall elastic properties have been demonstrated to have beneficial effects on probiotic bacteria strains. Bacteria with more deformable wall possess higher resistance to intracellular digestion by macrophages and a higher level of their activation [110]. Furthermore, microgels might be applied to mimic cell-cell interactions in a controlled microenvironment if isolated cell surface receptors (or their active parts) and the corresponding ligands can be embedded within the microgel surface. This will emulate the biological, biochemical and biophysical properties of the native cellular microenvironment and allow to disentangle the contribution of specific receptor types in the overall adhesive properties of cells. Thus, better identification of essential cues involved in pathological processes will be achievable. Future research opens the way for biochemical and biophysical modifications of microgels, which will improve the way of mimicking the extracellular matrix, thus enabling to detection or control of the cellular response in the altered surrounding microenvironment.

## Figures and Tables

**Table 1:** Examples of bulk fabrication strategies to prepare microgels *.

Monomer and Crosslinker	Microgel Properties	Ref.
**Precipitation polymerization**		
EGDMA-PolyEGDMA Co-polymerization of Divinylbenzene-PolyEGDMA-co-DVB	Size: PolyEGDMA: 1.18–2.27 µm (PDI = 1.006–1.035). PolyEGDMA-co-DVB: 1.93-2–45 µm (PDI = 1.006–1.019).	[33]
Co-polymerization MAA-PEGMM, EDMA crosslinker	Size: 0.45–3.25 µm depending on reactant conc. 0.77 µm (0.44%)–2.79 µm (20.3%) depending on solvent.	[34]
PEG-DA, crosslinked using Tetra-thiol + UV light	Size: 1.76 ± 0.26 µm. PDI = 1.26 (*n* < 200).	[35]
Co-polymerization N-isopropylacrylamide (NIPPAm) and acrylic acid (Acc), crosslinked using BIS (0.1,1 and 10 wt%)	Size: 2 ± 0.5 µm.	[36]
Co-polymerization PVA -PEGMMA, crosslinked using PEGDMA (1–11%) and HEMA4L46 (3%)	Size after sieving between 315 and 500 µm: PEGDMA: 376 ± 1 µm to 462 ± 2 µm. HEMA4L46: 379 ± 2 µm. Elastic modulus (G’): PEGDMA: 17±1 kPa to 1.1 ± 0.1 kPa. HEMA4L46: 4.8 ± 0.2 kPa.	[28]
**Surfactant-free emulsion polymerization**		
N-isopropylacrylamide, crosslinked using *N*,*N*’-methylenebisacrylamide	Size: 700 nm (room temp), 300 nm (310 K), PDI = 7%. Young‘s modulus (E): 8 ± 1–4 kPa (300 K), 86 ± 22 kPa (313 K).	[30]
Co-polymerization N-isopropylacrylamide (NIPPAm) and acrylic acid (Acc) crosslinked using BIS (0, 2, 4 and 7%)	Size: 760.3 ± 14.7 nm to 821.8 ± 47.0 nm depending on BIS concentration.	[37]
**Seed-feed**		
Co-polymerization MMA, MAA, EGD and GM Microgels: M-EGD, GM-E-EGD and DX microgels	Size: GM-M-EGD:102 nm (C.V 16) and M-EGD:107 nm (C.V 18). Storage modulus (G’): DX microgel storage modulus: 72.9–134 kPa.	[38]
**Spray drying**		
AcGGM Microgel: Hemicellulose	Size: 2.0 ± 1.0 µm (pure), 1.3 ± 0.3 µm, 2.7 ± 2.2 µm depending on added functional material.	[39]
Chitosan core and Eudragit coat	Core size: 1.8 ± 1.1 µm to 2.9 ± 1.7 µm depending on solvent and chitosan type. Core-coat size: 152 ± 4 µm to 223 ± 6 µm. Depending on Eudragit type and core/coat ratio.	[40]
**Membrane emulsification**		
Chitosan	Size: 12.92 (C.V 19.70%)–17.40 µm (C.V 21.94%) depending on monomer concentration. 13.81 (13.35%)–13.83µm (22.85%) depending on oil phase.	[41]
**Particle replication in nonwetting templates** (PRINT®)		
HEA PEGDA (1–10%)	Disc size: 5.2–5.9 µm d + 1.22-1.54 µm tall. Elastic modulus: 1% PEGDA: 7.8 ± 1 kPa 10% PEGDA: 63.9 ± 15.7 kPa.	[29]

* Abbreviations: AAc—acrylic acid; AcGGM—O-acetyl-galactoglucomannan; BIS—*N,N*’-methylenebisacrylamide; DVB—dvinylbenzene; DX—doubly crosslinked; EDMA—ethylene dimethacrylate; EGD—ethyleneglycol dimethacrylate; EGDMA—ethyleneglycol dimethacrylate; GM—glycidyl methacrylate; HEA—2-hydroxyethyl acrylate; HEMA4L46—ethylene glycol-co-tetralactic-co-tetraglycolic dimethacrylat; MAA—methacrylic acid; MAA—methacrylic acid; MMA—methyl methacrylate; NIPPAm—*N*-isopropylacrylamide; PEGDA—poly(ethylene glycol) diacrylate; PEG-DA—poly(ethylene glycol)-diacrylate; PEGDMA—poly(ethylene glycol dimethacrylate); PEGMM—poly(ethylene glycol) methyl ether methacrylate; PEGMMA—poly(ethylene glycol methyl ether methacrylate); PVA—poly(vinyl alcohol).

**Table 2:** Examples of microfluidic-assisted microgel fabrication processes *.

Polymer	Microfluidic Device	Sample Properties	Crosslinking	Properties of Microgels;	Ref
Alginate	Flow focusing for alginate emulsification, T-junction of Ca emulsification	η = 48 mPas at c_p_ = 1.5%	Ca induced. Coalescence between emulsified alginate and Ca droplets. Constant pH.	Non-spherical microgels with size in the range 150–100 µm Ø depending on flow rates. CV of size: 6.4%. Disc-like hydrogels.	[42,43]
	Flow focusing	Pronova UP MVG, Novamatrix c_p_ = 2.0%	Ca induced. Emulsified alginate with Ca-EDTA, Ca released from EDTA by acetic acid diffusing from oil phase after emulsification.	Spherical microgels with size in the range 17–50 µm Ø depending on flow rates. pH 5 in microgels.	[50]
	Flow focusing	Alginate, c_p_ = 2%	Ca induced. Emulsfied alginate with CaCO_3;_ increasing Ca solubility from the carbonate by acetic acid diffusing from the continuous phase. pH reduced	Nearly spherical microgels with size in the range 54–72 µm Ø obtained by varying flow rates.	[51,52]
	Flow focusing of dual inlet aqueous solutions	M_w_ = 268 kDa F_G_ = 0.68; used at c_p_ = 0.8%	Ca induced by competitive ligand exchange from different chelators at constant pH (user controlled in the range from 8 to 5).	Spherical micgrogels with size 50 µm Ø	[49]
					
κ-carra-geenan	Flow focusing	M_w_ = 1000 kDa, Copenhagen Hercules c_p_ = 0.8%;	Ca induced by including CaCl_2_ in the continuous phase (0.25% CaCl_2_ in undecanol).	Spherical microgels with size about 65 µm Ø obtained at given flow rates.	[53]
Pectin	Flow focusing	M = (239.5 ± 10.5) kDa; 24% amidation, 23% esterification; c_p_ = 1–10%	Ca induced; 4M CaCl_2_ dispersed at ratio 1:3 in mineral oil with 2 wt% span 80.	Spherical microgels with Ø~70µm and CV of 3.5% obtained under some conditions.	[48]
Gelatin		Calf bone gelatin, 300 Bloom; c_p_ of 5% in water at pH 7.4	Thermosetting: Emulsified by extrusion through microchannel plate, at 40 °C and collected in continuous phase as 25 °C, subsequently cooled to 5 °C.	Spherical microgels with diameter 31.6 µm, relative S.D. of 7.3%.	[54]
Hyaluronic acid derivatives	Flow focusing for initial emulsification of aqueous pregel, crosslinking initiator added in downstream junction	Hyaluronic acid derivatives. HA base polymer Mw 40–65 kDa	Passerini type X-linking using PEG dialdehyde initiated by compound added in the second junction; diffusing initiator from continous phase.	Spherical microgels wih tunable size, the range 70–90 µm diameter shown explicitly.	[20]

* Symbols used: η—viscosity; c_p_—polymer concentration; M—molar mass; M_w_—weight-average molar mass; F_G_—fraction of α-L-GulA in alginate (the other sugar residue is β-D-ManA); CV relative to variation in size.

**Table 3:** Examples of determination of microgel mechanical properties determined by AFM *.

Microgel Properties	Immobilization	Cantilever	AFM Mode	Shear or Young’s Modulus	Ref.
PNIPAm at c_p_ 18.2 mg/mL–21.8 mg/mL crosslinked with BIS	Adsorption on silicon substrate	MLCT-Bio-DC 0.01 N/m 0.03 N/m pyramid	FV; PF-QNM	3–32 kPa 17–48 kPa	[61]
Sodium alginate at c_p_ 0.9–1.7%, crosslinked with Ca or Ba ions; size range 425–870 µm	Micropatterned grid	MLCT 0.03 N/m–0.1 N/m pyramid	FV	0.4–14.4 kPa	[65]
(PNIPAM-co-PMAA/PDADMAC)9/PNIPAM-co-PMAA^b^ at c_p_ = 12 mmol, crosslinked with BIS. Size range 200–1400 nm	Adsorption: APTES coated silicon	SNML 0.07 N/m pyramid	PF-QNM	75 kPa at 25 °C 450 kPa at 40 °C	[70]
PNIPAm-co-Aac at c_p_ 3.7% and 5.4%, crosslinked with BIS, size range 280–480 nm	Adsorption on Au coated silicon	HQ:CSC38/NO AL 0.05 N/m cone	Microrheology	100 kPa–800 kPa	[72]
PNIPAm at cp 1.14% crosslinked either with 2.5 or 10 mol% BIS. Size of microgels ~1000 nm	Adsorption on Au coated silicon	AR-iDrive-N01 0.09 N/m pyramid	FV	1–4 kPa (for 2% BIS) 6–40 kPa (for 10% BIS)	[74]
MMA; size of the order ~100 nm	GOPS coated borosilicate	ScanAsyst-Fluid 0.4–0.7 N/m pyramid	PF-QNM	qualitative-deformation images	[75]
Alginate microspheres (UPLVG, FMC) at c_p_ = 1.5% crosslinked with Ca. Size larger than 100 µm	Deposition on nylon mesh; 330 µm holes; glued to Petri dish	PNP- TR 0.32 N/m pyramid	QI, scan size 3µm × 3µm	up to 8.75 MPa	[76]
Alginate microspheres (UPLVG, FMC) at c_p_ = 1.5% crosslinked with genipin. Size larger than 100 µm	Deposition on nylon mesh; 330 µm holes; glued to Petri dish	PNP-TR 0.32 N/m pyramid	QI, scan size 3µm × 3µm	up to 3.67 MPa	[76]
PNIPAm crosslinked with BIS; size of microgels 150–350 nm	Adsorption: APTES coated silicon	MLCT-BIO-DC 0.03 N/m pyramid	QI	16–140 N/m	[8,77]
Polyacrylamide (PAAm) 5.9% to 11.8% Crosslinked with BIS; size of microgels 13.3–18.0 µm	Plasma cleaned silicon	MLCT 0.03 N/m pyramid	FV	0.09–11 kPa	[17][
PAAm functionalized with NHS (N-succinimidyl ester) 7.9%, crosslinked with BIS–	Plasma cleaned silicon	MLCT 0.03 N/m pyramid	FV	1.6 kPa	[17]
pS-co-NIPAM, 50% to 70%; crosslinked with BIS (0–3 mol%); size of microgels 220–627 nm;	Not specified	silicon nitride 0.2–0.8 N/m	FV	0.24–0.99 GPa	[78]
ULC microgels—PNIPAm 146 mM Crosslinked with BIS; size range of microgels 1.1 ± 0.1 µm	Adsorption on APTES coated glass	silicon nitride 0.09 N/m sphere	FV	~10 kPa	[12,79]
Covalently crosslinked hyaluronic acid-based microgels fabricated using microfluidics. Size: 73–91 µm depending on crosslinker and c_p_	Adsorption on PEI imine coated, plasma cleaned glass	CSC38 0.09 N/m tipless	Force-distance	E from 11 to 34 kPa depending on sample	[20]

* Abbreviations used in the table: PNIPAm—poly(*N*-isopropylacrylamide); pS-co-NIPAM—poly(styrene-co-*N*-isopropylacrylamide); PVCL—poly(*N*-vinylcaprolactam; TBCHA—4-tert-butylcyclohexyl acrylate; *co*-PMAA—*co*-methacrylic acid; PDADMAC—poly-(diallyldimethylammonium chloride); PEG-PLPs—oligo ethylene glycol methacrylates with 4–5 ethyleneoxide repeating units and methacrylic acid; MMA—methacrylic acid; APTES—3-aminopropyl)triethoxysilane; GOPS—3-glycidoxypropyltrimethoxysilane; PBS—phosphate buffered saline; PAH—poly(allylamine hydrochloride); BIS—*N*,*N*’-methylene-bis-acrylamide; AAc—acrylic acid; FV—force volume; PF-QNM—peak force quantitative nanomechanics; QI—quantitative imaging; PNIPAM-co-PMAA microgel/PDADMAC multilayer films.
